# Tracheal Hamartoma: A Case Report

**DOI:** 10.7759/cureus.32128

**Published:** 2022-12-02

**Authors:** Mahfoud Imane, Sara Zarrouki, Meriem Rhazari, Afaf Thouil, Rachid Marouf, Hatim Kouismi

**Affiliations:** 1 Department of Respiratory Diseases, Mohammed VI University Hospital, Faculty of Medicine and Pharmacy, Mohammed First University, Oujda, MAR; 2 Department of Thoracic and Cardio-vascular Surgery, Mohammed VI University Hospital, Faculty of Medicine and Pharmacy, Mohammed First University, Oujda, MAR; 3 Department of Respiratory Diseases, Mohammed VI University Hospital, Oujda, MAR; 4 Research and Medical Sciences Laboratory (LRSM), Faculty of Medicine and Pharmacy, Mohammed First University, Oujda, MAR

**Keywords:** tracheal tumor, tracheal hamartoma, inspiratory dyspnea, rare benign tumor, pulmonary hamartoma

## Abstract

Hamartoma is the most frequently observed benign lung tumor, but its tracheal form is still exceptionally encountered. Cough, dyspnea, hemoptysis, and chest pain are all possible symptoms of tracheal hamartoma. The non-specific symptoms may also lead to a delayed diagnosis, and while the choice of treatment varies depending on the size and location of the lesion, conservative treatments remain strongly recommended. This report presents the case of a 57-year-old male who presented to our department with inspiratory dyspnea. Clinico-radiological data and bronchoscopy revealed a benign tracheal tumor of the lipomatous hamartoma type. The patient underwent a tumor resection by rigid bronchoscopy with satisfactory clinical results.

## Introduction

Primary tracheal tumors are rare, and only 10% are benign [1]. Although hamartomas are the most frequently observed benign pulmonary tumors, the tracheal form of this condition is exceptionally rare, especially in adults [2]. According to the literature, about ten cases of tracheal hamartoma have been reported [3]. Their rarity makes them underrecognized, which delays their diagnosis and thus therapeutic management [1,4]. We report a case of this scarce entity that was diagnosed based on the histological study of a surgical fragment.

## Case presentation

A 57-year-old man with a 30-pack-year of smoking presented with gradually progressive dyspnea (stage II of MMRC) associated with chronic dry cough and asthenia for one year. He had no family history of cancer, and no night sweats or known contact with a tuberculosis patient were reported. He had, however, a history of chronic alcohol consumption that he had withdrawn 30 years ago. We add that before the patient's visit to our department, he was first put on long-acting bronchodilators at an outside hospital for one year without any clinical improvement.

**Figure 1:**
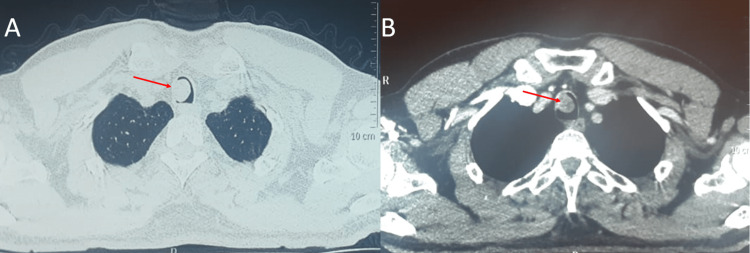
Thoracic CT scan, parenchymal (A) and mediastinal window (B), showing a round hypodense lesion within the trachea (red arrows).

Upon admission, the patient was stable and in a good general state with a 116/72 mmHg blood pressure, an 85 bpm heart rate, and a room air saturation of 98%. Furthermore, we found no reportable hemoptysis or weight loss upon investigation. Inspiratory stridor was found on pleuropulmonary examination. The rest of the physical examination found no significant abnormalities. The chest X-ray was performed but did not show any abnormalities. The CT scan showed a hypodense lesion in the proximal part of the trachea, obstructing almost the entire tracheal lumen (Figure 1).

**Figure 2:**
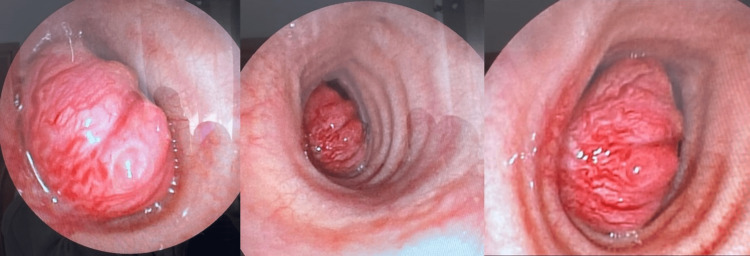
Bronchial fibroscopy images (from different angles) showing a budding lesion obstructing more than 90% of the tracheal lumen.

Bronchial fibroscopy revealed a bud obstructing more than 90% of the lumen. Six fragments were obtained during the bronchial biopsy, with results showing a non-specific inflammatory pattern without histological signs of malignancy (Figure 2). A rigid bronchoscopy under general anesthesia for diagnostic and therapeutic purposes was performed. The tumor was located 5 cm from the vocal cords. We went for resection by drilling with the placement of a tracheal prosthesis.

The anatomopathological study of the surgical specimen was in favor of a lipomatous tracheal hamartoma. The clinical evaluation was favorable, with a clear clinical improvement for our patient. He underwent respiratory functional explorations (RFEs) with satisfactory results. The patient is actually under regular follow-up, and the removal of the prosthesis was scheduled for three months after the surgery.

## Discussion

The first introduction of the term hamartoma was by Albrecht in 1904, referring to a benign tumor that consists of cells derived from primitive connective tissue such as cartilage, fat, bone, and smooth muscle cells [1,5,6].

Hamartomas are the most common benign tumors of the lung [7]. Only 1.4-10% of hamartomas are endobronchial, while intratracheal hamartomas are exceptionally rare [1,8]. In a retrospective study by Cosio BG et al. which included 71 hamartomas, tracheal localization was reported in only 2 cases [9]. They are 2 to 4 times more frequent in men, especially in the 6th decade of age [10,11]. Accordingly, our case is a male patient, aged 57 years.

The histological studies of endobronchial hamartomas show mainly adipose tissue; cartilage is not present or is present in very small quantities [9]. Which makes a histological diagnosis on small fragments obtained by bronchial biopsy difficult, as it is the case with our patient.

The clinical manifestations of tracheal hamartoma result from mechanical obstruction of the tracheal lumen [12,13]. They vary depending on the degree of luminal obstruction, ranging from inspiratory dyspnea to acute respiratory distress [12,13]. The diagnosis is often delayed because of the slow and silent evolution of the disease, essentially in patients with a history of smoking and chronic lung disease, where chronic symptomatology is usually linked with chronic obstructive pulmonary disease (COPD) or asthma [2,14]. Our patient had a history of progressive inspiratory dyspnea for 1 year. Following the worsening of his clinical condition after treatment with long-acting bronchodilators, central airway obstruction was suspected, and a thoracic CT scan was performed.

The endoscopic view of tracheal hamartoma presents as a polypoid lesion [15] with an inflammatory surface, which makes it macroscopically indistinguishable from a bronchogenic carcinoma [1]. Histological study on a large surgical resection specimen often seems necessary to retain the diagnosis of hamartoma.

The treatment of benign tracheal tumors varies according to the size and location of the lesion. Conservative treatments are often recommended, such as endobronchial electrocoagulation, laser therapy, and cryotherapy. However, surgical resection should be considered in cases where the tumor invades the tracheal wall [16]. In our case, the decision was to proceed with the resection of the tumor by rigid bronchoscopy under general anesthesia.

## Conclusions

Tracheal hamartomas remain underdiagnosed as they are often missed by physicians due to the variety of their symptoms. Despite being the most frequent benign tumors of the lung, they are unusual and relatively rare. The choice of treatment varies depending on the size and location of the lesion, but conservative treatments remain the first choice. Surgical resection should be considered in cases where the tumor invades the tracheal wall. Our work aims to highlight the diagnostic challenge of tracheal hamartoma as well as the importance of early management in preventing fatal complications.
